# Pregnancy and Birth Trends Across Australia, the United States of America and the United Kingdom

**DOI:** 10.3390/jcm14165841

**Published:** 2025-08-18

**Authors:** Anya L. Arthurs, Jade K. Harrison, Jessica M. Williamson, Claire T. Roberts

**Affiliations:** 1 College of Medicine and Public Health, Flinders University, Bedford Park, Adelaide, SA 5042, Australia; 2 Flinders Health and Medical Research Institute, Flinders University, Bedford Park, Adelaide, SA 5042, Australia; 3 School of Biomedicine and Robinson Research Institute, University of Adelaide, Adelaide, SA 5000, Australia

**Keywords:** pregnancy, birth, trend, delivery, obstetric, preeclampsia, gestational diabetes, preterm birth

## Abstract

Over the past two decades, pregnancy and birth trends have undergone significant shifts across Australia, the United States of America (USA), and the United Kingdom (UK), reflecting changes in societal norms, healthcare advancements, and demographic patterns. Variations in maternal age, birth interventions, and fertility rates highlight the evolving nature of reproductive behaviors and healthcare systems in these nations. The analysis reveals consistent increases in maternal age and gestational diabetes, alongside rising caesarean section rates—particularly in private healthcare settings. While perinatal mortality has declined overall, maternal mortality has increased in the USA and remains disproportionately high among Indigenous women and those in ethnic minorities in all three countries. These findings highlight the influence of structural inequities, healthcare access, and policy differences in maternal health. The review underscores the urgent need for equity-focused, culturally safe, and system-level interventions, as well as improved data collection and international collaboration to reduce preventable maternal and neonatal harms. By comparing these three regions, this review aims to provide insights into the shared challenges and unique approaches shaping childbirth practices in high-income countries in the 21st century.

## 1. Introduction: Birth Rates

This review examines evolving trends in pregnancy and birth outcomes across three high-income countries—Australia, the United States of America (USA), and the United Kingdom (UK)—drawing on data spanning from 1998 to 2024. The analysis encompasses a range of maternal and neonatal health indicators, including fertility and birth rates, maternal age, smoking during pregnancy, gestational diabetes mellitus (GDM), hypertensive disorders of pregnancy (HDP), preterm birth, fetal growth restriction, perinatal and maternal mortality, and mode and place of delivery. The manuscript highlights significant demographic and healthcare shifts, such as increasing maternal age, declining fertility, rising rates of medical intervention, and persistent ethnic and geographic disparities. By comparing these trends over the past two decades, the review aims to identify both shared challenges and context-specific issues that inform maternal health policy and practice in high-resource settings.

Data reported here are synthesized from publicly available national datasets and peer-reviewed literature to compare pregnancy and birth trends. A targeted search strategy was employed using databases such as PubMed, Scopus, and Google Scholar, as well as gray literature from verified and reputable sources including government and institutional repositories (e.g., Australian Institute of Health and Welfare, Centers for Disease Control and Prevention, Office for National Statistics). Search terms included combinations of “maternal health,” “birth trends,” “pregnancy complications,” “stillbirth,” “preterm birth,” “gestational diabetes,” and “hypertensive disorders of pregnancy,” filtered by country and year. Inclusion criteria encompassed national population-based reports and peer-reviewed studies published in English between 2000 and 2024 that provided data on pregnancy outcomes, maternal health indicators, or delivery practices. Sources were excluded if they lacked national-level data, were not verified or peer-reviewed, focused on low- or middle-income countries, or failed to stratify outcomes by maternal characteristics. The most recent and comprehensive data available as of early 2024 were prioritized to ensure relevance and accuracy.

Across Australia, the United States of America (USA) and the United Kingdom (UK; comprising England and Wales only for the purpose of this review) crude birth rates (live births per 1000 population) and total fertility rates (TFRs) continue to decline.

In Australia in 2021, there were 309,996 births registered and a crude birth rate of 12.1 [1]. This maintains the gradual decline in crude birth rate in Australia which was 13.6 in 2012 and 11.6 in 2022. Multiple births comprised 1.4% of mothers giving birth [2]. The TFR in 2022 was 1.63, a decrease from the 2002 TFR of 1.76 [2]. First Nations (Indigenous Australian and Torres Strait Islander) mothers comprise approximately 5% of all mothers in Australia [2], and in 2022, their babies comprised 5.3% of all births.

These trends are similar in the USA statistics. In 2022, 3,667,758 births were registered, with a crude birth rate of 11.0 which maintained a decline from a crude birth rate of 12.6 in 2012 [3]. Multiple births comprised 3.2% of all births. The TFR in 2022 was 1.67, a decrease from the 2002 TFR of 2.02 [4]. Notably, both are below the replacement TFR of 2.1. Indigenous American and Alaska Native mothers comprise approximately 0.7% of all mothers in the USA [5].

Similarly, in 2022 in the UK, 605,479 live births were registered, with a crude birth rate of 10.0. This maintained a decline from a crude birth rate of 12.8 in 2012; 1.5% of all births were multiple births. The total fertility rate in 2022 was 1.49, a decrease from the 2012 fertility rate of 1.92 [6].

Across Australia [2], the USA [4] and the UK [6], in 2021 and 2022, 49% of all babies born were female and 51% male.

## 2. Maternal Mortality

Maternal mortality is still a cause for concern in the 21st century, with 2020 statistics showing that a mother dies every two minutes worldwide [7]. Approximately 95% of these maternal deaths occurred in low and lower middle-income countries; sadly, the World Health Organization determined that most of these maternal deaths could have been prevented. The majority (~87%) of global maternal deaths were concentrated in sub-Saharan Africa and Southern Asia, with sub-Saharan Africa alone accounting for 70%.

The maternal mortality rate (MMR) describes the number of maternal deaths while pregnant per 100,000 live births. A promising trend has emerged over the 20-year period between 2000 and 2020, with reductions in MMR across the globe. The most significant reduction was from an MMR of 38 to 11 in Eastern Europe, and from 408 down to 134 in Southern Asia. Sub-Saharan Africa also experienced a reduction in MMR by 33%. However, the disparity in MMR is staggering when comparing low-income countries (MMR of 430 in 2020) with high-income countries (MMR of 13 in 2020), providing a clear indictment of our lack of equity in global healthcare during pregnancy.

Worldwide, three quarters of maternal deaths are due to haemorrhage and infection (usually postpartum), hypertensive disorders of pregnancy, delivery complications and unsafe abortion [8]. Across Australia [2], the USA [9] and the UK [10], the most common cause of maternal death that was not directly a result of pregnancy was cardiovascular disease (excluding COVID-19), with thromboembolism and thrombosis being the leading cause of maternal death directly related to pregnancy. Although the United Nations Millennium Development Goal aimed for a 75% reduction in maternal mortality from 1990 to 2015, for many high-income countries this has not been the case, including in the USA and UK. When ranked by highest to lowest MMR for 185 countries, the USA is ranked at #123, with the UK at #144 and Australia at #179 for 2020 [7].

Ethnicity still plays a significant role in maternal health. The Australian 2021 total MMR was 5.8, similar to that in previous years, 5.8 in 2020 and 5.7 in 2019. However, when stratified by Indigenous Australian status, the MMR for Indigenous mothers is a shocking 16.8 compared with 5.3 for non-Indigenous mothers [2]. The USA 2021 total MMR was 32.9, which was significantly increased compared to previous years MMR of 23.8 in 2020 and 20.1 in 2019. When differentiated by maternal ethnicity, the MMR for American Indigenous and Alaska Native mothers is 80.0 and for Black mothers is 69.9, 2.6- to 3-fold higher than that of White (26.6) and Hispanic mothers (28) [9]. Furthermore, the UK 2020-2022 total MMR was 13.4, an increase on the 2017–2019 MMR of 8.8. In a similar trend to that in the USA, maternal mortality in the UK was 3.8-fold more likely for Black mothers and 1.8-fold more likely for Hispanic mothers compared with White mothers [11].

### 2.1. COVID-19 Pandemic Effect on Maternal Mortality

It is likely that this increase in USA and UK maternal deaths in 2021 is due to the impact of COVID-19 on their respective healthcare systems [12]. In the USA, maternal mortality rates surged during the early years of the pandemic, disproportionately affecting Black and Hispanic women. This increase was attributed not only to direct viral impacts but also to disruptions in healthcare services, such as reduced access to prenatal care and overwhelmed hospital systems.

In Australia, while there were no maternal deaths directly linked to COVID-19 infection in 2020 or 2021, the pandemic led to significant changes in maternity care practices. These included a reduction in face-to-face antenatal visits, shorter postnatal hospital stays, and increased rates of hypertension during pregnancy. Such alterations may have long-term implications for maternal health, particularly concerning the management of pregnancy-related complications and mental health support [13,14].

The UK experienced a notable rise in maternal mortality during the pandemic, with COVID-19 becoming the leading cause of maternal death between 2019 and 2021. Indeed, the UK reported that the MMR for COVID-19-related maternal deaths between 2020 and 2022 was 1.9 [10]. The pandemic also exacerbated existing disparities, with Black women being four times more likely to die during or up to six weeks after pregnancy compared to White women. These outcomes highlight systemic vulnerabilities within maternal health services, including inadequate training for healthcare providers in managing complex cases and insufficient support for women with multiple disadvantages [15,16].

Collectively, these findings underscore the necessity for resilient and equitable maternal health systems. There is an urgent need for policies that ensure continuous access to comprehensive prenatal and postnatal care, address systemic inequities, and bolster mental health support for pregnant and postpartum individuals. Investments in culturally safe care practices and robust data collection are essential to mitigate the long-term impacts of the pandemic on maternal health outcomes.

It is noteworthy that the USA MMR is consistently higher than that of the UK or Australia, inconsistent with the norm for a high-income country. This could be due to a number of factors, including racial inequality and the strict laws against abortion in many USA states.

### 2.2. Reproductive Rights and MMR in the USA

The ethical and policy dimensions of reproductive rights are increasingly relevant to maternal health outcomes in the USA, particularly in the wake of the 2022 Dobbs v. Jackson Women’s Health Organization decision, which overturned Roe v. Wade. This legal shift has led to the implementation of restrictive abortion laws in multiple states, significantly limiting access to comprehensive reproductive healthcare. These restrictions not only infringe upon individual autonomy and bodily integrity but also carry direct implications for maternal morbidity and mortality. Evidence suggests that states with more restrictive abortion laws also tend to have higher maternal mortality rates and poorer access to maternity services, particularly for low-income and racially marginalized populations [17,18]. The ethical consequences of such policies are especially acute for individuals facing pregnancy complications where abortion may be a medically indicated intervention. Furthermore, these laws disproportionately affect women already experiencing structural disadvantage, amplifying existing inequities in maternal health. From a public health and human rights perspective, access to safe and legal abortion should be considered a critical component of equitable maternal healthcare, and its erosion poses significant risks to maternal wellbeing and reproductive justice [19].

## 3. Pregnancy

### 3.1. Maternal Age

Recent data from Australia, the USA and the UK reveal significant shifts in maternal age demographics and associated outcomes.

In Australia, both average maternal age (including mothers of all parities) and average first-time maternal age (including only primiparous mothers) are reported. The average maternal age has increased from 28.9 years in 1998 to 31.1 years in 2021 [1]. The average maternal age for First Nations mothers was lower than the national average but increased from 24.9 years in 2005 to 26.7 years in 2021. The national average first-time maternal age continues to rise, from 28.3 years in 2010 to 29.8 in 2022. Notably, the proportion of teenage mothers (under 20 years) has more than halved, from 3.7% in 2011 to 1.5% in 2021 and the proportion of First Nations teenage mothers (aged under 20) has decreased from 19% in 2011 to 10% in 2021. The rate of spontaneous labor onset among teenage mothers has declined from 68% in 2011 to 53% in 2021, with a corresponding increase in induced labor (from 27% in 2011 to 40% in 2021) [1]. Mothers aged 35 and over accounted for 26% of all births in 2021, a proportion that has remained relatively stable since 2011. These older mothers demonstrated high rates of early antenatal care access (81% in the first trimester) and frequent antenatal visits (96% having five or more visits). However, they also showed an increasing trend towards no labor onset (36% in 2021, up from 30% in 2011) and higher caesarean section rates (48% in 2021) [1]. The proportion of First Nations mothers attending antenatal care in the first trimester increased from 50% in 2012 to 72% in 2021, while those attending five or more antenatal visits rose from 85% to 88% during the same period [2]. Regarding birth methods, around 60% of First Nations mothers had a non-instrumental vaginal birth, while 33% gave birth by caesarean section [2].

The average maternal age in the USA of first-time mothers has also been steadily increasing over the past few decades, with significant implications for maternal and neonatal outcomes. The average age of first-time mothers in the USA was 27.54 years in 2023, an increase from 26.02 years in 2013, representing a rise of 1.52 years over a 10-year period. However, it is important to note that this figure presents the average age for first-time mothers only, not the average maternal age for all births [4] (for which data could not be located). Similar to Australia, birth rates in the USA differed according to different age subgroups. The birth rate for teenagers aged 15–19 continued to decline, dropping by 2% from 2021 to 2022, following a 7% decrease from 2020 to 2021 [4]. Contrastingly, the birth rate for women aged 40–44 rose by 5% in 2022, continuing an upward trend observed in previous years.

The average maternal age of first-time American Indigenous and Alaska Native mothers has been increasing, from 22.1 years in 2013 to 23.9 years in 2021, although this remains far below the national average. This trend is recapitulated in Black mothers, with an average maternal age for mothers having their first babies increasing from 23.1 years in 2013 to 25.5 years in 2021. Birth rates for teenage mothers (aged 15–19 years) continued to decline for Black and American Indigenous and Alaska Native women (down by 7% for both groups from 2021 to 2022) [4]. Sadly, American Indigenous and Alaska Native women are the least likely demographic group to access adequate prenatal care (7.3% inadequate) [20].

A 2023 large multi-center study of 302,484 multiparas in the USA revealed that caesarean delivery rates increased with maternal age, from 6.7% among 25-30 year olds, rising continuously to over 20% among those aged 43 years and older [21]. In 2022, 32.1% of all births in the USA were by caesarean delivery. When stratified by race, 36.8% of all births to Black mothers were by caesarean delivery; 35% of all births among 25–30 year-old Black mothers were by caesarean delivery, rising to 54.7% among those aged over 40 [4].

The trend of increased average maternal age in the past few decades is also observed in the UK. In 2022, the average maternal age for all births (including mothers of any parity) was 30.9 years, which has increased from 2010, where the average age was approximately 29.0 years [6]. The number of births by mothers under 20 years of age continues to decrease (from 5.6% in 2010 to 2.4% in 2022) and births by mothers over 40 years of age continue to increase (from 3.8% in 2010 to 5.2% in 2022). In addition, in 2020, the average age of first-time mothers in England and Wales was 29.1 years, which was the highest average age reported since the 1930s during the Great Depression. This trend has continued to increase with most women having their first child at 32 years of age as reported in 2024 [6].

These data show a clear trend for women in Australia, the USA and the UK having children later in life compared with previous generations. These findings underscore the evolving landscape of maternal age and its implications for obstetric care and outcomes.

### 3.2. Smoking During Pregnancy

In an encouraging trend, fewer women are smoking cigarettes during pregnancy across Australia, the UK and USA. In Australia in 2021, the rate of women smoking during pregnancy was 8.7% (compared with 13% in 2011). Mothers aged over 35 years were the least likely to smoke during pregnancy, with only 5.5% reporting that they smoked at any time while pregnant in 2021 [2]. Conversely, teenage mothers exhibit the highest smoking rates during pregnancy which comprised 33% of teenage mothers in 2021, although this rate has declined since 2011 during which 36% of teenage mothers reported that they smoked during pregnancy [1]. Encouragingly, the proportion of First Nations mothers smoking in the first 20 weeks of pregnancy fell from 50% in 2011 to 40% in 2021, and after 20 weeks of pregnancy from 45% to 36%.

In the USA, the percentage of mothers who smoked cigarettes during pregnancy declined to 4.6% in 2021, compared with 7.2% in 2016, with a decrease observed across all maternal age groups [22]. Similarly, the percentage declined across all race and Hispanic origin groups, and in all 50 states, along with the District of Columbia [22]. These data suggest a widespread reduction in smoking during pregnancy across the USA. However, there were age-related variations in smoking prevalence. Women aged 20–24 had the highest smoking prevalence during pregnancy at 10.7%, compared to 8.5% of women aged 15–19 (second highest prevalence) and 8.2% of women aged 25–29 (third highest prevalence). The lowest smoking prevalence was among women aged over 45 in whom 2% reported smoking, and women younger than 15 years of age whereby 2.5% reported smoking in 2018 [22,23].

The UK has also shown a reduction in cigarette smoking prevalence during pregnancy with 7.3% of women reported smoking in 2023 in comparison to 8.8% reported in 2022 [24]. This 1.5% drop marks the largest single-year decrease in smoking prevalence during pregnancy in the UK since the data collection began in 2006. Moreover, the percentage of women smoking during pregnancy has been steadily decreasing since 2006/2007 when approximately 16% of women smoked during pregnancy [24]. Comparable to Australia and the USA, younger women in the UK had a higher smoking prevalence during pregnancy with 28% of mothers aged under 20 smoking whilst pregnant in 2021/2022. In 2020, the 20–24 age group consistently had the highest prevalence of smoking during pregnancy and the 15–19 age group had the second highest prevalence [25]. These smoking prevalence trends according to specific age groups mirror the prevalence trends in the USA, whereby the lowest smoking prevalence was also seen in women younger than 15 years of age and older than 45 years of age [25].

These statistics highlight the overall reduction in smoking prevalence during pregnancy across Australia, the USA and the UK. However, the degree of reduction is age-group specific and younger mothers are at higher risk of smoking during pregnancy. Encouragingly, overall trends indicate positive changes in maternal health behaviors and outcomes among Australian First Nations women and ethnicities across USA, though disparities persist, highlighting the need for continued focus on culturally appropriate healthcare and support services.

### 3.3. Gestational Diabetes Mellitus (GDM)

Gestational diabetes mellitus (GDM) rates have been increasing globally in recent years. Here, we report the available statistics on GDM prevalence. However, it is worth noting that statistics should not be compared between countries, since in 2014, Australia instituted routine screening for GDM risk via an oral glucose tolerance test taken between 24 and 28 weeks’ gestation [26], whereas in the USA and UK, oral glucose tolerance tests for GDM risk are only administered where risk factors are identified. This likely means that many cases of GDM are undetected in the USA and UK, and that Australian data will report higher incidence.

There are differences in diagnostic criteria between Australia, the USA and UK, which may further explain the variability in prevalence. Since 2014, Australia has diagnosed GDM in accordance with the International Association of Diabetes and Pregnancy Study Groups (IADPSGs) recommended guidelines, which are consistent with the World Health Organization (WHO) guidelines. These diagnostic criteria use a 75 g oral glucose load with a GDM diagnosis made when any of the following is present: fasting plasma glucose ≥5.1–6.9 mmol/L, 1 h glucose ≥10.0 mmol/L, or 2 h glucose ≥8.5–11.0 mmol/L. Importantly, glucose levels exceeding these fasting and 2 h ranges result in a diagnosis of pre-existing diabetes mellitus, rather than GDM [27].

There is less uniformity in the USA [28]. GDM diagnosis can be as described above, in accordance with IADPSG criteria, using a 75 g oral glucose load. Alternatively, the American Diabetes Association (ADA) endorses the Carpenter and Coustan criteria [29]. This uses a 100 g oral glucose load and specifies GDM diagnosis at fasting glucose of 5.3 mmol/L, 1 h glucose 10.0 mmol/L, 2 h glucose 8.6 mmol/L, and 3 h glucose 7.8 mmol/L.

The UK diagnoses GDM in accordance with the National Institute for Health and Care Excellence (NICE) guidelines, which require fasting plasma glucose levels of ≥5.6 mmol/L or 2 h plasma glucose levels ≥7.8 mmol/L in response to 75 g oral glucose load, with no 1 h glucose level specified [30].

In Australia, GDM prevalence nearly doubled from 8.3% in 2014 to 16.3% in 2021, with rates rising significantly with maternal age, reaching 25.2% among mothers aged 40 and over [1]. First Nations mothers experienced a moderate increase in GDM incidence, from 9.3% in 2014 to 15% in 2021. Similarly, in the USA, GDM cases increased by 20% from 234,847 in 2016 to 281,789 in 2020, with the rate rising 30% from 6.0% to 7.8% over the same period [31]. This increase was particularly pronounced between 2019 and 2020, with a 9% rise compared to the average annual increase of 3% from 2016 to 2019. GDM rates varied significantly among ethnic groups, with Asian Indian women having the highest rate at 16.7%, followed by Vietnamese and Filipino women at 15.2% each. Among Hispanic subgroups, Mexican women had the highest rate at 8.9%. The prevalence of GDM increased with maternal age, from 2.5% for women under 20 to 15.3% for those 40 and over, and with plurality, from 7.7% for singleton births to 13.6% for triplets or higher-order multiples [31]. In England, 7.59% of deliveries in NHS hospitals in 2017–2018 were complicated by diabetes in pregnancy, with Asian women 2.9 times more likely, Black women 1.8 times more likely, and Chinese/Other women 1.7 times more likely to be affected compared to the general population [32]. These trends highlight the growing public health concern of GDM and the need for targeted interventions, especially among high-risk ethnic groups and older mothers.

### 3.4. Preeclampsia and Hypertensive Disorders of Pregnancy (HDP)

Hypertensive disorders of pregnancy (HDP) remain a significant global health concern, with varying trends observed across different regions. Hypertensive disorders of pregnancy include preeclampsia, gestational hypertension (GHTN), eclampsia, and haemolysis, elevated liver enzymes, and low platelet count (HELLP) syndrome. HDPs indicate blood pressure elevation during gestation or postpartum, and account for ~7.4% of pregnancy-related deaths [2,33,34].

In Australia, gestational hypertension is defined as a new onset of hypertension, after 20 weeks of gestation, with systolic >140 mmHg or diastolic >90 mmHg. These guidelines were adopted in 2014 by the Society of Obstetric Medicine of Australia and New Zealand (SOMANZ) [35]. These guidelines were updated in 2023, although the definition of hypertension in pregnancy remained unchanged. Preeclampsia diagnosis occurs when gestational hypertension is paired with an additional indicator of other organ damage, such as proteinuria, elevated serum creatinine (>90 μmol/L), neurological involvement or pulmonary oedema. The current USA guidelines were introduced by the American College of Obstetricians and Gynecologists (ACOGs) in 2013. In the UK, the NICE guidelines were used for both GHTN and preeclampsia, since 2013 [36,37]. Both the ACOG and NICE guidelines are consistent with the Australian guidelines for both the diagnosis of GHTN and preeclampsia.

Preeclampsia is considered a potentially life-threatening HDP as it affects 2–8% of pregnancies worldwide manifesting as hypertension with proteinuria or other organ dysfunction in the second half of pregnancy [38]. Importantly, preeclampsia alone is responsible for around 14% of maternal deaths globally [33] and is a major cause of perinatal morbidity and mortality.

Two decades of research have documented an association between preeclampsia and major cardiovascular disorders in later life [7,34,39,40,41,42]. Analysis confirmed that preeclampsia and HDP almost double the risk of subsequent cardiovascular events, with preterm preeclampsia leading to an even higher risk [43]. As expected, women who had preeclampsia were more likely to be nulliparous, diabetic, hypertensive, overweight or obese, and less likely to smoke cigarettes. Pregnancies affected by preeclampsia were more likely to be delivered preterm compared to those without it and had a lower mean infant birthweight. In addition, preeclampsia and HDP almost double the risk of a subsequent cardiovascular event and preterm preeclampsia inflates that risk [33,39,43].

In Australia, gestational hypertension (GHTN) has remained relatively stable at approximately 3.2% since 2014, with a higher prevalence in first pregnancies (4%) compared to second pregnancies (2.2%) [2]. Unfortunately, no reports on preeclampsia incidence in Australia outside of the broader GHTN classification could be found. An incidence of preeclampsia increased in First Nations mothers, from 2.8% in 2014 to 3.4% in 2021, while GHTN as a broader classification remained at a prevalence of ~3.3%.

In the USA, HDP prevalence among births in hospitals increased from 13.3% to 15.9% between 2017 and 2019, with the highest rates observed in women aged 35–44 (18.0%) and 45–55 years (31.0%), as well as among Black women (20.9%) and American Indian and Alaska Native women (16.4%) [42]. Notably, 31.6% of maternal deaths occurring during delivery hospitalization had a diagnosis code for HDP documented [42]. A comprehensive analysis of USA data revealed a significant increase in HDP overall incidence from 2.79% in 1989 to 8.22% in 2020, representing an average annual percentage change of 3.6% [43]. Chronic hypertension also increased (average annual percent change 4.1%), while eclampsia rates decreased (average annual percent change −2.5%) [44].

In the UK, a population-based cohort study of 1.3 million women from 1997 to 2016 found that 2.42% of women experienced preeclampsia, with 76.64% of cases occurring in the first pregnancy [34]. Moreover, in NHS hospitals in England (2017–2018), gestational diabetes prevalence was 6.76%, with Black women approximately twice as likely to be affected, while eclampsia rates were 0.07%.

### 3.5. Preterm Birth (PTB) and Fetal Growth Restriction (FGR) Leading to Low Birthweight

Globally, preterm birth (PTB) and low birthweight rates have remained relatively stable, with approximately 8.2% and 6.3%, respectively, in 2021 [45,46]. However, region-specific rates have varied in recent years.

Preterm birth is defined by the WHO as a baby born alive before 37 weeks of gestation, and this definition is consistently adopted in Australia, the USA and the UK [47].

In Australia, the PTB rate stands at approximately 8.6%, translating to more than 26,000 babies born preterm each year [2]. This rate is slightly lower than the global average of one in ten babies being born early [46]. Notably, the PTB rate among First Nations women is significantly higher at 13.8%, compared to 8.4% for non-First Nations mothers [2]. The Northern Territory reports the highest rates of PTB in the country, with a rate of 11.4% in 2020, highlighting ongoing disparities in maternal health outcomes [2]. These statistics emphasize the need for culturally appropriate care and targeted interventions to improve maternal and neonatal health across different populations. FGR, defined by the Australian Institute of Health and Welfare as a baby born with a birthweight less than the third centile for gestational age and sex, decreased from 3.4% of births (at or after 40 weeks’ gestation) in 2004 to 2.0% in 2022 [2].

In the USA, the early PTB rate declined from 2.14% in 2019 to 2.11% in 2020 [48]. The overall percentage of all PTBs in the USA decreased from 10.49% in 2021 to 10.38% in 2022, following a 4% rise from 2020 (10.09%) to 2021 [49]. Specific data for FGR could not be sourced.

In England and Wales, 7.8% of all live births were preterm in 2022, with rates in Wales slightly higher at 8.1% compared to England’s 7.8% [34]. Low birthweight (<2.5 kg) rates at term were reported as 2.86% across the UK, with Wales at 2.5% and England at 2.9%. Data from NHS hospitals in England for 2017–2018 indicated a preterm birth rate of 6.32%, with similar likelihood across ethnicities [34]. These figures highlight the persistent global challenge of preterm births and low birthweight, emphasizing the need for continued efforts to improve maternal and neonatal health outcomes.

### 3.6. Perinatal Deaths and Stillbirth

Perinatal mortality rates vary significantly across different countries and demographic groups. In Australia, 2021 data revealed 9.6 perinatal deaths per 1000 births, including 7.1 per 1000 stillbirths [50]. Stillbirth in Australia is defined as a baby born with no signs of life that weighs more than 400 g, or more than 20 weeks in gestation. Of the 3016 perinatal deaths, three-quarters were stillbirths, with congenital anomalies being the most common cause. Notably, the rate of stillbirth for First Nations pregnancies shows an upward trend from 8.3 stillbirths per 1000 births in 2015 to 13.1 in 2021, whilst the national stillbirth rate did not significantly change over the same period. However, the number of pregnancies for First Nations women over this period increased 1.5-fold, which may explain this discrepancy [51].

In the USA, the Centers for Disease Control and Prevention defines stillbirth as the loss of a baby after 20 weeks’ of gestation. The fetal mortality rate has shown a gradual decline from 7.49 per 1000 births in 1990 to 5.73 per 1000 births in 2021 [52]. However, significant racial disparities persist, with Black non-Hispanic, American Indian or Alaska Native non-Hispanic, and Native Hawaiian or other Pacific Islander non-Hispanic populations experiencing substantially higher rates compared to White non-Hispanic and Asian non-Hispanic groups [52].

In England and Wales, stillbirths are defined as babies born after 24 or more weeks’ completed gestation and which did not, at any time, breathe or show signs of life [53]. There were 2433 stillbirths in 2022, a 6.3% decrease from 2021, with rates of 3.9 per 1000 live births in England and 4.4 per 1000 in Wales [53]. While the overall stillbirth rate in these countries decreased to 4.0 per 1000 total births in 2022, it remains higher than pre-pandemic levels, underscoring the ongoing challenges in reducing perinatal mortality.

### 3.7. Birth Location and Skilled Support

Almost all registered births took place in hospitals. In Australia in 2022, 97% of all births were in conventional labor wards in hospitals, with 75% of these in a public hospital. Additionally, 2% of mothers chose to deliver in a birth center and 0.5% at home [2]. In the USA in 2017, 98.4% of registered births took place in hospitals, with 0.99% choosing to deliver at home and 0.52% in birth centers [54]. In the UK, where birth centers are a more common option for delivery, 97.6% of registered births in 2022 took place in a National Health Service (NHS) establishment (encompassing hospitals and maternity care units), with 0.3% occurring in non-NHS (private) establishments and 1.9% of births occurring at home [6].

Regardless of their place of delivery, ~99% of all births in Australia, the USA and UK benefited from the presence of a skilled medical professional, be that a midwife, doctor or nurse. Compared with only 68% of births in low income, and 78% in lower-middle income countries which are attended by skilled health personnel [55], it is not surprising that the maternal mortality rate (MMR) differs so strongly between countries [7].

### 3.8. Mode of Delivery

Overall, birthing trends are similar between Australia [2] and the UK [34]. The majority of women gave birth after spontaneous labor, although this rate had decreased compared with previous years. The rate of women giving birth via caesarean section continues to increase.

In Australia in 2021, 50% of women had a non-instrumental vaginal birth, down from 56% in 2011. The rate of instrument-assisted vaginal births (including vacuum and forceps) was 12.1%, remaining steady since 2011. The rate of births via caesarean section was 38%, a marked increase from 32% in 2011 [2]. Similarly, in the UK in 2022, the majority of women (47.8%) gave birth via non-instrumental vaginal birth, a significant decrease from 57.7% in 2017. There was a modest decrease in instrument-assisted vaginal births, at 11.2% in 2022 from 12.3% in 2017. The rate of caesarean sections significantly increased in recent years, to 38.5% in 2022 from 28.4% in 2017 [34].

In the USA in 2022, 67.8% of women had a vaginal birth (unspecified as to whether instrument assisted or not), and 32% of women delivered via caesarean section. Both rates have remained relatively steady since 2012, with no differences in delivery method with ethnicity reported [3].

**Figure 1:**
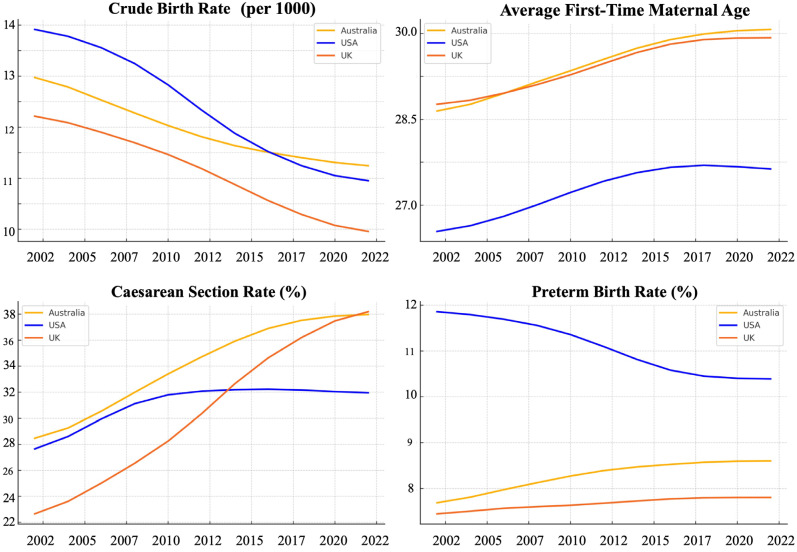
Smoothed Trends in Maternal and Perinatal Indicators across Australia, the USA and the UK between 2002 and 2022.

While the rising caesarean section rates are well-documented, particularly among older mothers and those giving birth in private hospitals, these trends have sparked ongoing debates about what constitutes appropriate medical intervention in childbirth. In many cases, caesarean delivery can be life-saving when clinically indicated, for example, in cases of fetal distress, obstructed labor, or severe preeclampsia. However, growing evidence suggests that rates above 10–15% at the population level are not associated with further reductions in maternal or neonatal mortality [56]. Rates exceeding this threshold may reflect over-medicalization rather than improved outcomes, especially when driven by non-medical factors such as provider convenience, fear of litigation or financial incentives in fee-for-service systems. Critics argue that excessive reliance on surgical delivery undermines physiological birth processes, increases maternal morbidity and contributes to longer recovery times and complications in subsequent pregnancies [57]. Others contend that the focus should not be on reducing caesarean section rates per se, but rather on ensuring that the decision to intervene is grounded in shared decision-making, respectful maternity care, and individualized risk assessment. Addressing this complex issue requires policies that promote appropriate, evidence-based use of intervention while resisting one-size-fits-all targets that may inadvertently compromise safety or autonomy (Table 1; Figure 1).

## 4. Breastfeeding

Breastfeeding rates vary significantly across different countries and demographic groups. In Australia, the 2020–2021 National Health Survey (NHS) reported that 96% of infants aged 0–3 years were breastfed at least once [2]. However, breastfeeding rates declined as infants aged, with 80% receiving breast milk at 4 months, 74% at 6 months, and 51% at 12 months. Moreover, the National Aboriginal and Torres Strait Islander Health Survey (NATSIHS) revealed that among First Nations infants aged 0–3 years, only 11.7% were exclusively breastfed to 6 months and continued to 12 months, while 7.5% were exclusively breastfed for at least 6 months but less than 12 months, and 80.6% were not exclusively breastfed for at least 6 months [2]. In the UK, 81% of term deliveries in 2017–2018 had skin-to-skin contact, and 74% of babies received breast milk as their first feed [34]. The USA reported that 83.2% of infants born in 2019 were breastfed at least once, with 45.3% exclusively breastfed through 3 months and 24.9% through 6 months [58]. Notably, breastfeeding rates varied significantly by state, with Alabama, Louisiana, Mississippi, and Florida having the lowest percentages, while Alaska, Colorado, Idaho, and Washington were among the highest [58]. These disparities highlight the need for targeted interventions to improve breastfeeding rates, particularly among vulnerable populations.

Breastfeeding rates across Australia, the USA and the UK are shaped not only by maternal health behaviors but also by broader social, economic and policy factors. Paid parental leave, workplace flexibility and access to supportive healthcare services are among the most influential determinants of breastfeeding initiation and duration. For example, Australia and the UK offer publicly funded maternity leave programs, whereas the USA lacks a national policy for paid maternity leave which may contribute to its lower exclusive breastfeeding rates beyond three months compared with Australia and the UK [2,6,58]. Socioeconomic status and maternal education also play a critical role; women with lower income or education levels are less likely to initiate or continue breastfeeding, often due to job insecurity, limited access to lactation support, or the necessity of early return to work [2,58]. Cultural beliefs and partner support further influence breastfeeding practices, as do hospital policies and practices, including adherence to the WHO/UNICEF Baby-Friendly Hospital Initiative which has been shown to increase breastfeeding initiation [58]. Racial and ethnic disparities are well documented, particularly in the USA and UK, where Black and Hispanic women, as well as First Nations and Indigenous populations, often face systemic barriers such as reduced access to culturally safe health services, inadequate prenatal education and historical mistrust in healthcare systems [2,6,58]. These factors underscore the importance of addressing social inequities and strengthening structural supports to improve breastfeeding outcomes across diverse populations.

## 5. Discussion and Conclusions

While maternal age, smoking, GDM and HDP are discussed individually, these factors often interact in complex, causally linked ways. Most notably, advanced maternal age is an established independent risk factor for a range of pregnancy complications, including GDM, HDP, preterm birth and stillbirth. Age-related physiological changes such as reduced vascular elasticity, insulin resistance, and endothelial dysfunction may partially explain this increased risk. For instance, older maternal age is associated with decreased glucose tolerance and increased adiposity, contributing to higher rates of GDM [59]. Similarly, endothelial dysfunction and chronic inflammation, more prevalent in older women, contribute to the pathophysiology of preeclampsia and gestational hypertension [60]. In turn, these complications are themselves risk factors for preterm delivery and caesarean section, highlighting a cascading effect of risks initiated by delayed childbearing. Socioeconomic and lifestyle factors, such as higher BMI, comorbidities and differential access to care, may further compound these age-related risks. A more integrated approach to maternal health surveillance and management is needed to account for the interplay between these factors and to support risk reduction strategies, particularly as the trend toward later maternal age continues in high-income countries.

### 5.1. Limitations

While this review aims to provide a comprehensive cross-national comparison of maternal and perinatal health trends, several methodological limitations must be acknowledged. Direct comparisons between Australia, the USA and the UK are complicated by inconsistencies in data definitions, measurement methods, and reporting standards across countries. For instance, definitions of stillbirth vary by gestational age thresholds (≥20 weeks in Australia and the USA versus ≥24 weeks in the UK) and routine screening protocols for conditions such as gestational diabetes differ markedly. Australia employs universal screening, while the USA and UK use risk-based approaches, leading to substantial variation in reported prevalence. Additionally, reporting systems for maternal mortality and preterm birth rely on different classification criteria and levels of completeness, with some datasets lacking granularity by parity, ethnicity or maternal age. These discrepancies limit the extent to which data can be directly compared or aggregated and underscore the need for standardized global reporting frameworks in maternal health research. Despite these challenges, synthesizing available national data provides important insights into shared trends and population-specific disparities in high-income settings.

It is important to note that different years have been used for reporting pregnancy complication data throughout this review. This variation reflects the heterogeneous nature of data collection and publication timelines across different national health agencies and jurisdictions. For example, the most recent and complete datasets for gestational diabetes and hypertensive disorders of pregnancy vary by country, with some agencies releasing comprehensive annual summaries while others rely on periodic audits or hospital-based datasets. To ensure the most accurate reflection of current trends, the latest available data for each indicator was used, even if that meant reporting from slightly different time periods. Additionally, data comprehensiveness varies between countries. Australia benefits from a nationally standardized perinatal data collection managed by the Australian Institute of Health and Welfare (AIHW), whereas in the USA and UK, a mix of federal, state, and NHS datasets are used, some of which are stratified by race, region or care setting. While these differences may impact direct comparability, the selected data represent the best available evidence and provide valuable insights into maternal and perinatal health trends across high-income countries.

### 5.2. Contextualzsing Findings Within a Social Determinants Framework

To deepen the interpretation of our findings, we situate them within established theoretical frameworks that conceptualize reproductive health as shaped by broader social, economic and political forces. The World Health Organization’s Commission on Social Determinants of Health (CSDH) underscores the fact that inequities in health outcomes are largely driven by the conditions in which people are born, grow, live, work, and age and calls for action on structural drivers such as income inequality, education, healthcare access, and systemic discrimination [61]. These structural determinants help explain why Indigenous and minority women in high-income countries continue to experience higher rates of maternal mortality, preterm birth, and stillbirth, even after accounting for clinical risk factors [9,11,13]. The ecosocial theory, developed by Nancy Krieger, further highlights how social conditions “get under the skin,” by biologically embedding inequality through cumulative exposures across the life course and generations and providing a useful lens for understanding how chronic stress, racism and marginalization may increase the physiological risk of adverse pregnancy outcomes [62]. Additionally, the intersectionality theory, as articulated by scholars such as Kimberlé Crenshaw, provides a critical tool for understanding how multiple social identities, such as race, gender, class, and Indigeneity intersect to compound disadvantages in maternal healthcare settings [63]. For example, Indigenous mothers may face not only geographic and economic barriers to care but also culturally unsafe practices, racial profiling and medical mistrust that together undermine care quality and outcomes. Taken together, these frameworks emphasize the need for upstream policy action to address entrenched structural inequities in maternal health, complementing clinical and behavioral interventions with broader reforms focused on equity, justice and culturally safe care.

### 5.3. Policy Recommendations

This review highlights both shared and divergent trends in maternal and neonatal health across Australia, the USA and the UK over the past two decades. While progress has been made in areas such as reductions in smoking during pregnancy and improved perinatal survival, persistent inequities remain, particularly in maternal mortality, preterm birth and stillbirth rates among Indigenous and racial minorities. Based on the comparative analysis, several specific policy recommendations emerge.

First, all three countries would benefit from adopting national targets to reduce maternal mortality and stillbirth rates, with dedicated funding to support evidence-based interventions in underserved populations. Australia and the USA could learn from the UK implementation of continuity of midwifery-led care models, which are associated with improved maternal satisfaction and reduced intervention rates. Conversely, the UK and Australia should consider adopting the USA more granular collection and public reporting of maternal outcomes stratified by race, ethnicity and social determinants, enabling more targeted policy responses.

Governments should mandate the routine disaggregation of maternal and perinatal health data by ethnicity, geography and socioeconomic status and require annual public reporting to enhance accountability. Expansion of publicly funded, culturally safe perinatal programs, such as Australia’s Birthing on Country initiatives, should be prioritized, with adaptations to local Indigenous and community-led contexts in the USA and UK. In parallel, all three countries should implement anti-racism training and structural competency education across the maternity care workforce to address bias in clinical decision-making.

To reduce preventable caesarean sections, health systems should revise funding models that incentivize surgical delivery and instead support value-based maternity care that rewards high-quality, woman-centered outcomes. In the USA, studies have shown that hospitals with fee-for-service structures and for-profit status have higher caesarean rates, in part due to the higher reimbursement associated with surgical delivery [64,65]. Similarly, in Australia, caesarean section rates are significantly higher in the private sector, where obstetricians are remunerated per procedure, and elective scheduling of deliveries is more common [66,67]. This dynamic is less prominent in publicly funded systems like the UK NHS, where most maternity services are salaried and centrally funded, reducing financial drivers for surgical delivery. Additionally, universal paid parental leave, improved access to postpartum care (especially for those with GDM or HDP), and cross-sectoral policies targeting housing, income, and food security are needed to tackle the upstream determinants of poor pregnancy outcomes.

Finally, we recommend the creation of an international maternal health equity taskforce to facilitate knowledge exchange and coordinate progress toward reducing disparities globally. Through coordinated policy action and investment in equity-driven care models, countries can move beyond incremental improvements to achieve systemic change in maternal and neonatal health outcomes.

### 5.4. Conclusions

In conclusion, pregnancy and birth trends in Australia, the USA, and the UK demonstrate consistent patterns of declining fertility rates, increasing maternal age, and rising rates of medical intervention, particularly caesarean births. While all three countries have seen improvements in maternal behaviors such as reduced smoking during pregnancy and increased engagement with antenatal care, particularly among older mothers and some Indigenous and minority groups, disparities remain stark. Maternal mortality remains unacceptably high in the USA, especially for Black and American Indigenous mothers, while in Australia, First Nations mothers continue to experience significantly worse outcomes, including higher rates of maternal death, preterm birth and stillbirth resulting from a lack of culturally safe care and trauma associated with colonization. The increasing prevalence of gestational diabetes and hypertensive disorders of pregnancy, particularly among older mothers and ethnic minorities, reflects shifting clinical challenges that must be met with tailored screening, management and follow-up. These findings highlight the urgent need for culturally informed, equity-driven maternity care policies and targeted public health interventions to improve outcomes for vulnerable populations across all three nations.

## Figures and Tables

**Table 1:** Comparison of pregnancy and birth trends across Australia, the USA and the UK between 2021 and 2023.

Indicator	Australia	USA	UK
Crude Birth Rate (per 1000) (2022)	11.6	11.0	10.0
Total Fertility Rate (2022)	1.63	1.67	1.49
Multiple Births (%) (2022)	1.4	3.2	1.5
Maternal Mortality Rate (per 1000) (2021 or latest)	5.8	32.9	13.4
Average First-Time Maternal Age (years) (2022)	29.8	27.5	29.7
Teenage Mothers (%) (2021 or latest)	1.5	3.9	2.4
Smoking During Pregnancy (%) (2022)	8.7	4.6	7.3
Caesarean Section Rate (%) (2022)	38.0	32.0	38.5
Preterm Birth Rate (%) (2022)	8.6	10.38	7.8

## Abbreviations

The following abbreviations are used in this manuscript:

USA	United States of America
UK	United Kingdom
TFR	Total fertility rates
MMR	Maternal mortality rate
GDM	Gestational diabetes mellitus
HDP	Hypertensive disorders of pregnancy
GHTN	Gestational hypertension
HELLP	Haemolysis, elevated liver enzymes, and low platelet count syndrome
PTB	Preterm birth
FGR	Fetal growth restriction
NHS	National Health Service
